# Causal Structure Learning in Continuous Systems

**DOI:** 10.3389/fpsyg.2020.00244

**Published:** 2020-02-20

**Authors:** Zachary J. Davis, Neil R. Bramley, Bob Rehder

**Affiliations:** ^1^Department of Psychology, New York University, New York, NY, United States; ^2^Department of Psychology, The University of Edinburgh, Edinburgh, United Kingdom

**Keywords:** causal learning, dynamic systems, computational modeling, intervention, cognitive modeling, resource limitations

## Abstract

Real causal systems are complicated. Despite this, causal learning research has traditionally emphasized how causal relations can be induced on the basis of idealized events, i.e., those that have been mapped to binary variables and abstracted from time. For example, participants may be asked to assess the efficacy of a headache-relief pill on the basis of multiple patients who take the pill (or not) and find their headache relieved (or not). In contrast, the current study examines learning via interactions with continuous dynamic systems, systems that include continuous variables that interact over time (and that can be continuously observed in real time by the learner). To explore such systems, we develop a new framework that represents a causal system as a network of stationary Gauss–Markov (“Ornstein–Uhlenbeck”) processes and show how such *OU networks* can express complex dynamic phenomena, such as feedback loops and oscillations. To assess adult's abilities to learn such systems, we conducted an experiment in which participants were asked to identify the causal relationships of a number of OU networks, potentially carrying out multiple, temporally-extended interventions. We compared their judgments to a normative model for learning OU networks as well as a range of alternative and heuristic learning models from the literature. We found that, although participants exhibited substantial learning of such systems, they committed certain systematic errors. These successes and failures were best accounted for by a model that describes people as focusing on pairs of variables, rather than evaluating the evidence with respect to the full space of possible structural models. We argue that our approach provides both a principled framework for exploring the space of dynamic learning environments as well as new algorithmic insights into how people interact successfully with a continuous causal world.

## Introduction

We live and act in a messy world. Scientists' best models of real-world causal processes typically involve not just stochasticity, but real-valued variables, complex functional forms, delays, dose-dependence, and feedback leading to rich and often non-linear emergent dynamics (Cartwright, 2004; Strevens, 2013; Sloman and Lagnado, 2015). It follows that learning successfully in natural settings depends on accommodating these factors. Cognitive psychologists have explored many of these dimensions of complexity in isolation (e.g., *stochasticity:* Waldmann and Holyoak, 1992; Bramley et al., 2017a; Rothe et al., 2018; *interventions:* Sloman and Lagnado, 2005; Waldmann and Hagmayer, 2005; Bramley et al., 2015; Coenen et al., 2015; *time:* Buehner and May, 2003; Lagnado and Sloman, 2006; Rottman and Keil, 2012; Bramley et al., 2018; and *continuous variables:* Pacer and Griffiths, 2011). However, we argue these components generally can not be isolated in realistic learning settings, meaning a deeper understanding of human causal cognition will require a new framework that naturally accommodates inference from interventions in continuous dynamic settings.

As an everyday example of a time-sensitive, dose-dependent causal relationship, consider the complexities involved in consuming alcohol. It is common for drinkers to adjust their consumption based on their recognition that higher doses affect inhibition or mental clarity, that will in turn have other downstream effects on quality of conversation or willingness to sing karaoke. The effects of alcohol consumption differ widely in quality and quantity depending on dosage and time delays. A small glass of wine with dinner will likely have little effect on mental clarity whereas a few shots will have a stronger effect. Further complicating the learning problem, these effects of alcohol do not come instantaneously but are rather delayed and distributed in time. Worse still, there can be complex temporal dynamics, such as the feedback loop between lowered inhibition and increased alcohol consumption, and innumerable contributing factors, such as diet or amount of sleep, that modulate alcohol's effect. Thus, in settings like this, the learning problem is non-discrete (how much alcohol did I drink) and extended in time (when did I drink it), produces evidence that is naturally time ordered (how you feel over the preceding and subsequent hours), and involves complicated dynamics (e.g., feedback loops). In the current paper, we study human learning through real-time interactions with causal systems made up of continuous valued variables. We see this setting as capturing the richness of real world causal learning, while remaining simple and principled enough to allow for a novel formal analysis.

The structure of the paper is as follows. First, we summarize relevant past work on causal structure inference from interventions, temporal information, and different representations of functional form. Next, we lay out our new formalism for inference of causal structure between continuous variables. We then report on an experiment, in which participants interact with causal systems represented by sliders on the computer screen. We provide an exploratory analysis of the interventional strategies we observed in the experiment before analyzing structure learning through the lens of a normative Bayesian inference model and a range of heuristic and approximate alternatives, finding evidence that people focus sequentially on individual connections rather than attempting to learn across the full space of possible causal models at once. Finally, we discuss new opportunities provided by the formalism introduced in this paper, including future questions in causal cognition as well as applications to other areas, such as dynamic control.

### Past Research

#### Probabilistic Causation Over Discrete Events

**Figure 1:**
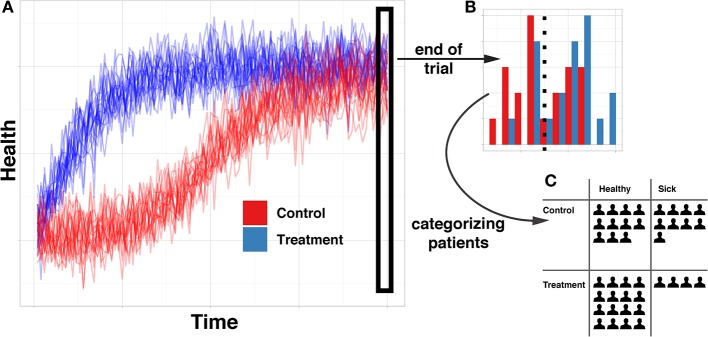
Illustration of abstraction from full timeseries data to probabilistic contingency. **(A)** is a full time course of the health of 40 simulated patients throughout the course of a classic randomized controlled trial. **(B)** demonstrates the type of information available when only evaluating the health of patients at the end of the trial. **(C)** demonstrates the type of information available when categorizing patients into “sick” and “healthy” groups, rather than maintaining full continuous information.

Research in causal cognition has generally aligned itself with the philosophical tradition of probabilistic causation, which defines a causal relationship as one where a cause changes the probability of its effect (Hitchcock, 2018). This definition implicitly operates over particular representations: discrete states, such as events or facts that have some probability of occurring or being true. Because of this, experimental work in causal cognition has primarily focused on causal relationships between discrete valued (often binary) variables (e.g., Sloman, 2005; Krynski and Tenenbaum, 2007; Ali et al., 2011; Fernbach and Erb, 2013; Hayes et al., 2014; Rehder, 2014; Rothe et al., 2018). These are typically presented in contexts in which temporal information is either unavailable or abstracted away so that cases can be summarized in a contingency table. See Figure 1 for a simple example in which (A) continuous data is (B) snapshotted in time, in order to (C) dichotomize and create counts of contingencies and ultimately abstracted into a probabilistic causal relationship. This approach is very common in part because there is a well-established mathematical framework—*Bayesian networks*—for efficiently encoding joint distributions of sets of variables in the form of networks of probabilistic contingencies (Pearl, 2009; Barber, 2012).

While the probabilistic contingencies paradigm has been fruitful for exploring many aspects of causal cognition, we are interested in other settings. As mentioned, we believe that many real life systems may not lend themselves to discretization, nor involve much independent and identically distributed data with no temporal information. Instead, people are often have access to autocorrelated, time-dependent, continuous information and we are interested in they how represent and draw inferences on the basis of this information.

#### Learning

A prominent question in causal cognition is how people learn causal relationships from contingency data, such as that presented in Figure 1C. Although the literature shows that humans are often quite adept causal learners (Cheng, 1997; Griffiths and Tenenbaum, 2005; Lu et al., 2008) there are a number of important exceptions. One is that updates to beliefs about causal structure on the basis of new information are often made narrowly rather than globally. That is, in ways that do not compare the evidential fit across all variables taken together. To model this, Fernbach and Sloman introduced a *Local Computations* (LC) model, which posits that people focus on “evidence for individual causal relations rather than evidence for fully specified causal structures” (Fernbach and Sloman, 2009, p. 680). By ignoring the possible influences of other causes, their model captures a strong empirical tendency for human learners to exhibit order effects and overconnect their causal hypotheses (also see Taylor and Ahn, 2012). Bramley et al. (2017a) extended this finding, finding evidence suggesting that people consider local changes that modify their previously favored hypothesis. Together, these studies suggest that people use a local updating strategy, testing and evaluating individual causal links rather than updating a posterior distribution over the global model space. We ask whether this tendency toward local learning extends to the continuous dynamic systems that are under study here.

#### Learning via Interventions

As well as capturing probabilistic relationships, Bayesian networks can be used to reason about, and from, idealized manipulations of causal systems, or “interventions” (Pearl, 2009). Bayesian networks, at their core, deal with *independence*, not dependence, relations. Because of this, if a cognizer passively observes some variables but cannot observe the temporal direction of their influences (i.e., perhaps they influence one another too quickly to see) they can be equally consistent with multiple causal hypotheses. For example, the common cause *X* ← *Y* → *Z* and chain *X* → *Y* → *Z* are “Markov equivalent” because, in both networks, *X* and *Z* are independent conditional on *Y*. However, crucially, Markov equivalent networks do not have identical data distributions under intervention. In the example of Markov equivalent networks given above, intervening to set *Y* to some value *y* as denoted with Pearl's (2009) “Do()” operator, would change the distribution for *X* under the common cause—i.e., *P*(*X*) ≠ *P*(*X*|Do[*Y* = *y*]) for at least some *y*—but would not affect the distribution for *X* for the chain—i.e., *P*(*X*) = *P*(*X*|Do[*Y* = *y*]) for any *y*.

It has been shown that people are able to learn successfully from interventions, and are often moderately efficient in their intervention selection according to information–optimal norms (Steyvers et al., 2003; Sloman and Lagnado, 2005; Waldmann and Hagmayer, 2005; Coenen et al., 2015; Bramley et al., 2017a). However, participants in these studies also typically exhibited biases indicative of the influence of cognitive constraints. For example, Coenen et al. (2015) found that, when deciding between two potential causal networks, people appeared to follow a heuristic of intervening on the node with the most downstream causal links (averaged across the candidate networks) rather than intervening to maximally distinguish between the two. Use of this heuristic was more common when intervening under time pressure. Bramley et al. (2017a) tested people's learning in a broader hypothesis space encompassing all possible 3 and 4 variable network structures. They found that people made interventions that appeared to target uncertainty about a specific individual link, node or confirm a single hypothesis, rather than those effective at reducing their uncertainty “globally” over all possible causal networks. Here we assess the efficacy of learners' interventions on continuous dynamic systems for which variables are potentially manipulated through a range of magnitudes over an extended period of time.

#### Time

Time has long been seen as a powerful cue for causation (Hume, 1959), especially with regards to identifying causal direction. People rule out backwards causation, assuming that effects cannot precede causes (Burns and McCormack, 2009; Greville and Buehner, 2010; Bramley et al., 2014). Work in the cognitive sciences on the use of time in causal judgments has focused on point events separated by delays—that is, events like explosions and collisions that occur at particular times but with negligible duration (Shanks et al., 1989; Griffiths, 2004; Lagnado and Sloman, 2006; Pacer and Griffiths, 2012; McCormack et al., 2015). From this line of work, we have learned more than just that temporal order is relevant for causal direction. The actual temporal dynamics of causal systems affect judgments, for example shorter and more reliable delays between cause and effect are more readily seen as causal (Greville and Buehner, 2010).

In a systematic study of people's use of temporal dynamics to learn causal structure, Bramley et al. (2017b, 2018) combined interventions and time to investigate people's learning of causal structure between components that exhibited occasional (punctate) events that could also be brought about by interventions. They found that people are sensitive to expected delays, especially when they also expect the true delays to be reliable, and are judicious and systematic in their use of interventions. While these studies have been valuable in demonstrating that people are sensitive to the temporal characteristics of causal systems, many everyday systems—such as economies, ecosystems, or social groups—are more naturally described as extended shifting influences than point events. We thus see the current study as extending the analysis of time's role in causal cognition to explore these inherently continuous settings.

#### Continuous Variables

As discussed above, many natural scenarios involve continuous valued variables and causal influences that are typically extended in time rather than punctate. Given the ubiquity of such systems, continuous variables have received surprisingly little attention in the study of causal cognition. In a reanalysis of data from Marsh and Ahn (2009) and a novel experiment, Pacer and Griffiths (2011) showed that people are capable of learning individual cause-effect relationships between continuous variables. Soo and Rottman (2018) investigated causal relations in non-stationary time series, i.e., those where long term trends affect the average values of the variables in ways that obscure and complicate the causal relations between those variables. They proposed three ways that the variables could be represented before assessing their relationships: (1) state values, (2) difference scores, and (3) trinarized difference scores (positive, negative, or zero). In their task, causal strength judgments were best explained by the correlation between the direction of *changes* in variables' values from one time point to the next, rather than direct correlation between the variables.

#### Complex Problem Solving

This project connects to the literature on complex problem solving (Berry and Broadbent, 1984)—also sometimes called complex dynamic control (Osman, 2010). This line of work explores goal-directed behavior in dynamic environments, typically with a structure that is hidden and initially unknown to participants. In particular, we follow Funke (2001) in studying minimal complex systems (MICS) that change dynamically in response to participants' actions and their hidden structure, but are not so complex as to prohibit formal analysis. MICS have been used as psychometric measurement tools, having been shown to provide individually stable and reliable predictors of real-world achievement (Greiff et al., 2013). This suggests that MICS tap into fairly foundational cognitive abilities.

Research on complex problem solving has begun to unpack the key features of such MICS, and of the cognitive strategies recruited by participants that determine performance. For example, when participants have narrow goals in a new environment, they learn less about its overall structure (Vollmeyer et al., 1996), a finding consistent with proposals that monitoring goals induces cognitive demands (Sweller, 1988). They are also less likely to engage in systematic strategies that can aid learning, such as the Vary One Thing At a Time (VOTAT, see Kuhn and Brannock, 1977; Tschirgi, 1980) or PULSE strategy (Schoppek and Fischer, 2017). Other work has identified a number of high level behavioral features, such as time on task, number of interventions made, or strategies, that predict likelihood of success (Greiff et al., 2016; Schoppek and Fischer, 2017; Stadler et al., 2019).

We build on previous work in the CPS literature in a number of ways. For one, whereas tasks in the CPS literature are typically self-paced, we are unusual (but not unique, see Brehmer and Allard, 1991; Schoppek and Fischer, 2017) in studying time-continuous systems. We take the task of reacting to dynamics as they unfold in real time to be reflective of real world dynamic control scenarios. More fundamentally, the research area's focus on predicting success in *control* has left a gap in our understanding of what exactly participants are *learning* as they interact with dynamic systems. The current work extends on this line of enquiry by providing a close model-based analysis of participants' actions and learning.

In sum, our approach here is novel in two key respects. First, we study a setting that, like reality, is continuous in terms of both time and state space. This allows us to study learning in the context of causal systems that give rise to non-linear emergent dynamics through the lens of a sophisticated normative and heuristic model comparison. Second, we explore an interactive setting in which participants intervene on the system of interest in complex, extended ways, rather than merely passively observing its behavior or setting states across discrete trials, again mapping more onto real world actions than the idealized interventions studied in much of the existing causal learning literature.

## The Task

We chose a simple and intuitive structure learning task interface that allows for learners to use their mouse to interact with the variables in a system represented by a set of moving sliders on the computer screen. A depiction of how the sliders were presented is shown in **Figure 4**. Participants could observe the evolving sequence of variable values but also move and hold the variables (one at a time) at positions of their choice by using the mouse. As mentioned, this environment allows us to test learning of causal systems with continuous valued variables and feedback dynamics. It also allows us to assess learning via interventions that are both extended over time (learners choose how long to intervene) and non-stationary (learners might “hold” the variable in a particular position or “wiggle” it up and down).

## Continuous Causality in Time

This section presents a formalism for modeling causal systems that relate continuous variables in time. To define a generative model for such systems, we first introduce the notion of an Ornstein–Uhlenbeck (OU) process and then define how multiple OU processes can be interrelated so as to form an interacting causal system. We then describe normative inference within this model class on the basis of both observational and interventional data.

### Generative Model

#### The Ornstein–UhlenbeckProcess

An Ornstein–Uhlenbeck (OU) process is a stationary Gauss-Markov process that reverts to a stable mean (Uhlenbeck and Ornstein, 1930). It can be conceptualized as Brownian motion with the addition of a corrective force that biases the process's expected value toward the mean of the distribution. The magnitude of that force increases as a function of the distance been that mean and the process's current state. Formally, $\Delta v_{i}^{t}$—the change in variable *i* from time *t* to *t* + 1—is defined as

(1) $$\begin{array}{l} P\left(\Delta v_{i}^{t}|\omega ,\mu_{i},v_{i}^{t},\sigma \right)=\omega \left[\mu_{i}-v_{i}^{t}\right]+N\left(0,\sigma \right) \end{array}$$

where $v_{i}^{t}$ is the value of *i* at time *t*, μ_*i*_ is the mean of the process for variable *i*, σ is its variance, and ω is a parameter > 0 that determines how sharply the process reverts to the mean^1^. μ_*i*_ is also referred to as the process's *attractor state* because it is the value to which the process will revert to at asymptote. See **Figure 3A** for an example of an OU process with an attractor state of 0.

#### OU Processes and Causality

This definition can be generalized to accommodate OU processes with non-stationary means. In particular, we take the step of assuming that the attractor state μ for a variable is determined by some function of the most recent values of its cause(s). When a variable has no causes we model its attractor state as being 0.

##### The single cause case

**Figure 2:**
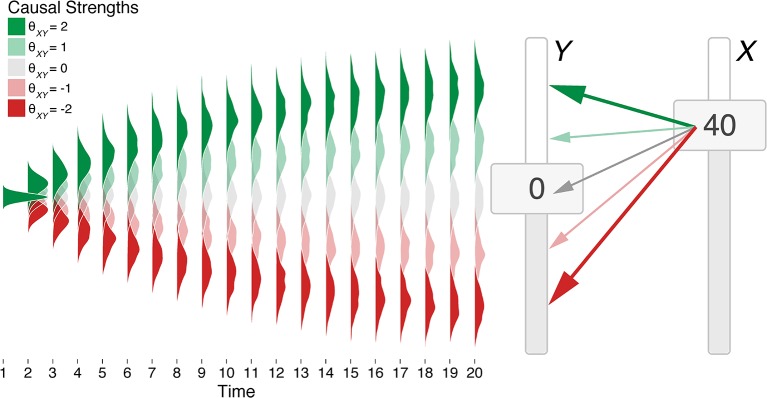
Visualization of the impact of a single cause (slider *X*) on a single effect (slider *Y*) in an OU network with different causal strengths. Slider *X* is held to a value of 40 for 20 timepoints, leading slider *Y* to unfold over time to different values depending on the causal strength. Probability distributions are smoothed averages of 100 runs of the network given different causal “strengths” θ_*XY*_ (colored shading) where ω = 0.1 and σ = 5.

For a variable *i* with a single cause *j* this function is simply,

(2) $$\begin{array}{l} \mu_{i}^{t+1}=f\left(v_{j}^{t}\right) \end{array}$$

where $v_{j}^{t}$ is the value of *j* at time *t*. As *j* changes over time, so too does the output of $f\left(v_{j}^{t}\right)$, which serves as the new attractor state of variable *i* at the next timepoint. For simplicity, here we assume that $f\left(v_{j}^{t}\right)$ is linear. Thus, the change in *i* at the next timestep ($\Delta v_{i}^{t}$) is

(3) $$\begin{array}{l} P\left(\Delta v_{i}^{t}|v_{i}^{t},v_{j}^{t},\omega ,\sigma ,\theta_{ji}\right)=\omega \left[\theta_{ji}\cdot v_{j}^{t}-v_{i}^{t}\right]+N\left(0,\sigma \right) \end{array}$$

where θ_*ji*_ ∈ (−∞, ∞) is a multiplier (or “strength”) mapping the value of the cause *j* to the attractor state of effect *i*. Figure 2 presents how a variable *Y* changes as a function of its cause *X* for a number of different values of θ_*XY*_. We assume Δ*t* of 100 ms (i.e., between *t* and *t* + 1) and that ω and σ remain constant, although these assumptions can be loosened (see Lacko, 2012).

##### The multiple cause case

In general, a variable may have more than one cause. Although there are a variety of ways in which multiple causal influences might combine (cf. Griffiths and Tenenbaum, 2009; Pacer and Griffiths, 2011), here we simply assume that causes have an additive influence on an effects' attractor state, such that

(4) $$P(\Delta v_{i}^{t}|v^{t},\omega ,\sigma ,\Theta )=\omega \left[[\sum_{j}\theta_{ji}\cdot v_{j}^{t}]-v_{i}^{t}\right]+N(0,\sigma )$$

where *j* now ranges over all causes of variable *i* and Θ is a square matrix such that θ_*ji*_ ∈ Θ is the strength of the causal relationship from *j* to *i*^2^. Simply put, the mean that variable *i* reverts to is assumed to be a sum of the values of its causes, each first multiplied by their respective θs.

A collection of connected OU processes, which we call an *OU network*, defines causal relationships for all directed relations between variables and unrolls these effects over time. For example, for a system consisting of variables *X*, *Y*, and *Z*, Θ specifies the strengths of the six potential inter-variable causal relationships: *X* → *Y*, *Y* → *X*, *X* → *Z*, *Z* → *X*, *Y* → *Z*, and *Z* → *Y*. Note that non-relationships are specified in this scheme by setting θ_*ji*_ to zero. At each timestep, Equation (4) is used to determine $v_{X}^{t+1}$, $v_{Y}^{t+1}$, and $v_{Z}^{t+1}$ as function of their previous values $v_{X}^{t}$, $v_{Y}^{t}$, and $v_{Z}^{t}$. For display purposes, it is sometimes necessary to constrain *v* to be between some range. This is done by setting all *v*^*t* + 1^ that fall outside of the range to their nearest value in the range. The clock then moves forward and the process repeats.

**Figure 3:**
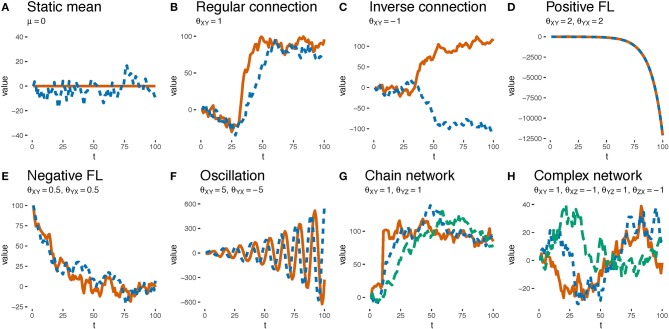
Examples of the dynamical phenomena resultant from varying θ weights. Solid red, dotted blue, and dashed green lines depict the values of variables *X*, *Y*, and *Z*, respectively. **(A)** A system with a single variable *Y* whose distribution mean is stationary at 0 (i.e., μ = 0). **(B)** A system with variables *X* and *Y* and a θ weight from *X* and *Y* of 1 (i.e., θ_*XY*_ = 1). μ_*X*_ = 0 for first 30 timepoints and then μ_*X*_ = 100 for next 70. The value of *Y* tracks the value of *X*. **(C)** The same as **(B)** except that *X* and *Y* are negatively related (θ_*XY*_ = −1). The value of *Y* tracks but has the opposite sign of *X*. **(D)** A system in which *X* and *Y* are reciprocally related via θ weights that are >1 (i.e., θ_*XY*_ = θ_*YX*_ = 2). Because the values of *X* and *Y* grow so large they are indistinguishable in the plot. **(E)** The same as **(D)** except that *X* and *Y*, which have an initial value of 100, are reciprocally related via θ weights that are <1 (θ_*XY*_ = θ_*YX*_ = 0.5). The values of *X* and *Y* eventually fluctuate around 0. **(F)** The same as **(D)** except that the reciprocal θs are large and of opposite sign (i.e., θ_*XY*_ = 5, θ_*YX*_ = −5). The values of *X* and *Y* oscillate. **(G)** A system with three variables whose θ weights form a causal chain, θ_*XY*_ = θ_*YZ*_ = 1. μ_*X*_= 0 for 10 timepoints but then is set to 100 via an intervention. Note that changes in *Y* precede changes in *Z*. **(H)** Timeseries of actual data observed by participant 10 on trial 10, generated by a complex system with three variables and four non-zero θs. All variables were initialized at 0 and there were no interventions.

OU networks have some intuitively appealing features of continuously varying causal relationships. Figure 3 demonstrates some of the dynamics that emerge from causal systems simply by varying the θs. Whereas, a positive θ_*XY*_ results in the value of *Y* following some positive multiple of the value of *X* (Figure 3B), a negative θ_*XY*_ means that a decrease in *X* drives up the value of *Y* (e.g., decreasing interest rates is generally thought to increase inflation, Figure 3C). Feedback loops are naturally represented with non-zero values of θ_*XY*_ and θ_*YX*_. A positive feedback loop results if the θs are of the same sign and have an average magnitude >1 (Figure 3D) whereas a negative feedback loop results if they are <1 (Figure 3E). Oscillations can be implemented with θs of mismatched signs (such as 5 and −5, Figure 3F). Such feedback loops can be implemented between pairs of variables or as part of a cyclic causal structure with potentially many variables. Combining feedback loops and cycles and including asymmetrical forms can lead to even more complex dynamics (e.g., Figure 3H). We invite the reader to build their own network and observe the dynamics at https://zach-davis.github.io/html/ctcv/demo_ctcv.html. Note that while the discussed examples cover two or three variables, the OU networks framework generalizes to any number of variables.

### Inference

We follow Griffiths and Tenenbaum (2005) in modeling people's learning of causal graphs as inverting the generative model. What must be inferred is the causal structure most likely responsible for producing all variable values at all timepoints—*v*—under interventions.

Note that to accommodate interventions, we adopt Pearl's (2009) notion of graph surgery. If variable *i* is manipulated at time *t*, the likelihood that $v_{i}^{t}$ has its observed value is 1 (i.e., is independent of *i*'s previous value or the value of its causes). We define $\iota_{i}^{t}$ as an indicator variable that is true if variable *i* is intervened on at *t* and false otherwise.

#### The Single Cause Case

Consider the inference problem in which the goal is to determine whether variable *j* causes variable *i*, and if so, the sign of that causal relationship. That is, assume a hypothesis space *L* with three hypotheses. One is that θ_*ji*_ is >0, a causal relationship we refer to as a *regular connection*. A second is that θ_*ji*_ is <0, referred to as an *inverse connection*. Finally, θ_*ji*_ = 0 denotes that *j* has no impact on *i*. Assume that *i* has no other potential causes.

Computing the posterior probability of a causal hypothesis *l*_*k*_ ∈ *L* involves computing, for each timepoint *t*, the likelihood of the observed change in *i* ($\Delta v_{i}^{t}$) given the previous values of *i* and *j* ($v_{i}^{t}$ and $v_{j}^{t}$), a value of θ_*ji*_ corresponding to the hypothesis, the endogenous system parameters ω and σ, and any intervention that may have occurred on *i* ($\iota_{i}^{t}$). If the learner did not intervene on *i* at *t*, this likelihood is given by Equation (3). If they have, it is 1. The product of these likelihoods over all timepoints is proportional to the posterior probability of *l*_*k*_.

(5) $$\begin{array}{l} P\left(l_{k}|v_{i},v_{j};\iota_{i}\right)\propto \underset{t}{\prod }\int_{\omega }\int_{\theta_{ji}}\int_{\sigma }P\left(\Delta v_{i}^{t}|v_{i}^{t},v_{j}^{t},\omega ,\sigma ,\theta_{ji};\iota_{i}^{t}\right)\\ P\left(\theta_{ji}|l_{i}\right)P\left(l_{k}\right)P\left(\omega \right)P\left(\sigma \right)\text{d}\sigma \text{d}\theta_{ji}\text{d}\omega \end{array}$$

*P*(ω) and *P*(σ) represents the learner's prior beliefs about ω and σ. *P*(θ_*ji*_|*l*_*k*_) represents the priors over θ_*ji*_ corresponding to hypothesis *l*_*k*_. For example, if *l*_*k*_ corresponds to a regular connection, *P*(θ_*ji*_|*l*_*k*_) would be 0 for non-positive values of θ_*ji*_. For positive values, it would reflect learner's priors over θ_*ji*_ for regular connections (later we describe how these priors can be estimated in our experiment on the basis of an instructional phase that precedes the causal learning task). Applying Equation (5) to each causal hypothesis and then normalizing yields the posterior over the three hypotheses in *L*.

A complication arises if variable values *v* are truncated between some range of values (in our task *v* ∈ [–100, 100]). In the case where $v_{i}^{t}$ equals the maximum truncated value, the likelihood is the mass of the likelihood distribution above the range of values. For the minimum truncated value the likelihood is the mass of the likelihood distribution below the range of values.

#### The Multiple Cause Case

This procedure for evaluating a single potential causal relationship generalizes to determining the structure of an entire OU network. Consider a hypothesis space *G* as consisting of *graphs* where each graph defines, for every potential causal relationship, whether it is positive, inverse, or zero. For a system with *n* variables *G* would contain 3^2*n*^ distinct causal hypotheses; for our example system with variables *X*, *Y*, and *Z*, *G* contains 729 graphs. The posterior probability of a graph *g*_*k*_ ∈ *G* involves computing for each variable *i* and timepoint *t*, the likelihood of the observed $\Delta v_{i}^{t}$ given the θs defined by *g*_*k*_ and the state of the system's variables at *t* (Equation 4), taking into account the possibility of an intervention on *i* at *t* ($\iota_{i}^{t}$):

(6) $$\begin{array}{l} P\left(g_{k}|v;\iota \right)\propto \overset{N}{\underset{i=1}{\prod }}\underset{t}{\prod }\int_{\omega }\int_{\theta }\int_{\sigma }P\left(\Delta v_{i}^{t}|v^{t},\omega ,\sigma ,\theta ;\iota_{i}^{t}\right)\\ P\left(\theta |g_{k}\right)P\left(g_{k}\right)P\left(\omega \right)P\left(\sigma \right)\text{d}\sigma \text{d}\theta \text{d}\omega \end{array}$$

## Experiment: Causal Structure Learning

To test people's ability to learn causal structure between continuous variables in continuous time, we conducted an experiment in which participants freely interact with sliders governed by an OU network with hidden causal structure. Their goal was to intervene on the system in order to discover the hidden causal structure.

### Method

#### Participants

Thirty participants (13 female, age *M* = 37.5, *SD* = 10.6) were recruited from Amazon Mechanical Turk using psiTurk (Crump et al., 2013; Gureckis et al., 2016). They were paid $4 for ~30 min. In a post-test questionnaire, on a ten point scale participants found the task engaging (*M* = 7.9, *SD* = 2.2) and not particularly difficult (M = 3.9, *SD* = 2.6). All procedures were approved by the Institutional Review Board of New York University (IRB-FY2016-231).

#### Materials

**Figure 4:**
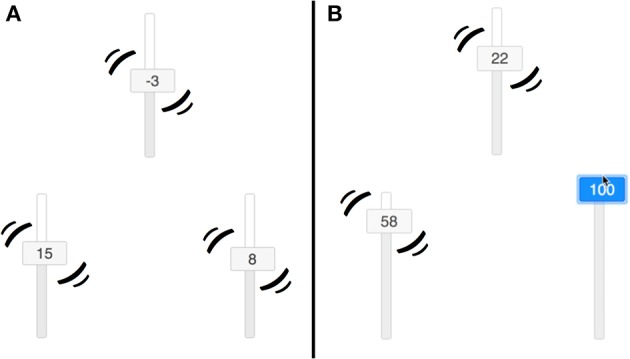
Sliders used by participants. **(A)** Shows that the sliders all jitter if no interventions are made. **(B)** Shows that the sliders do not jitter if intervened on.

Each of the three variables was represented by a vertical slider that moved by itself according to the underlying OU network but which could also be manipulated by clicking and dragging anywhere on the slider, overriding the state it would otherwise have taken (see Figure 4)^3^. A timer was presented at the top of the screen. Participants responded using six additional sliders presented beneath the trial window, one for each potential causal relations. Responses were constrained to be one of three options: “Inverted,” “None,” or “Regular,” corresponding to θ < 0, no relationship (θ = 0), and θ > 0, respectively. Participants were pre-trained on these terms in the instructions. The sliders were constrained to be between −100 and 100, and the buttons on the slider presented a rounded integer value in addition to moving up and down.

#### Stimuli and Design

**Figure 5:**
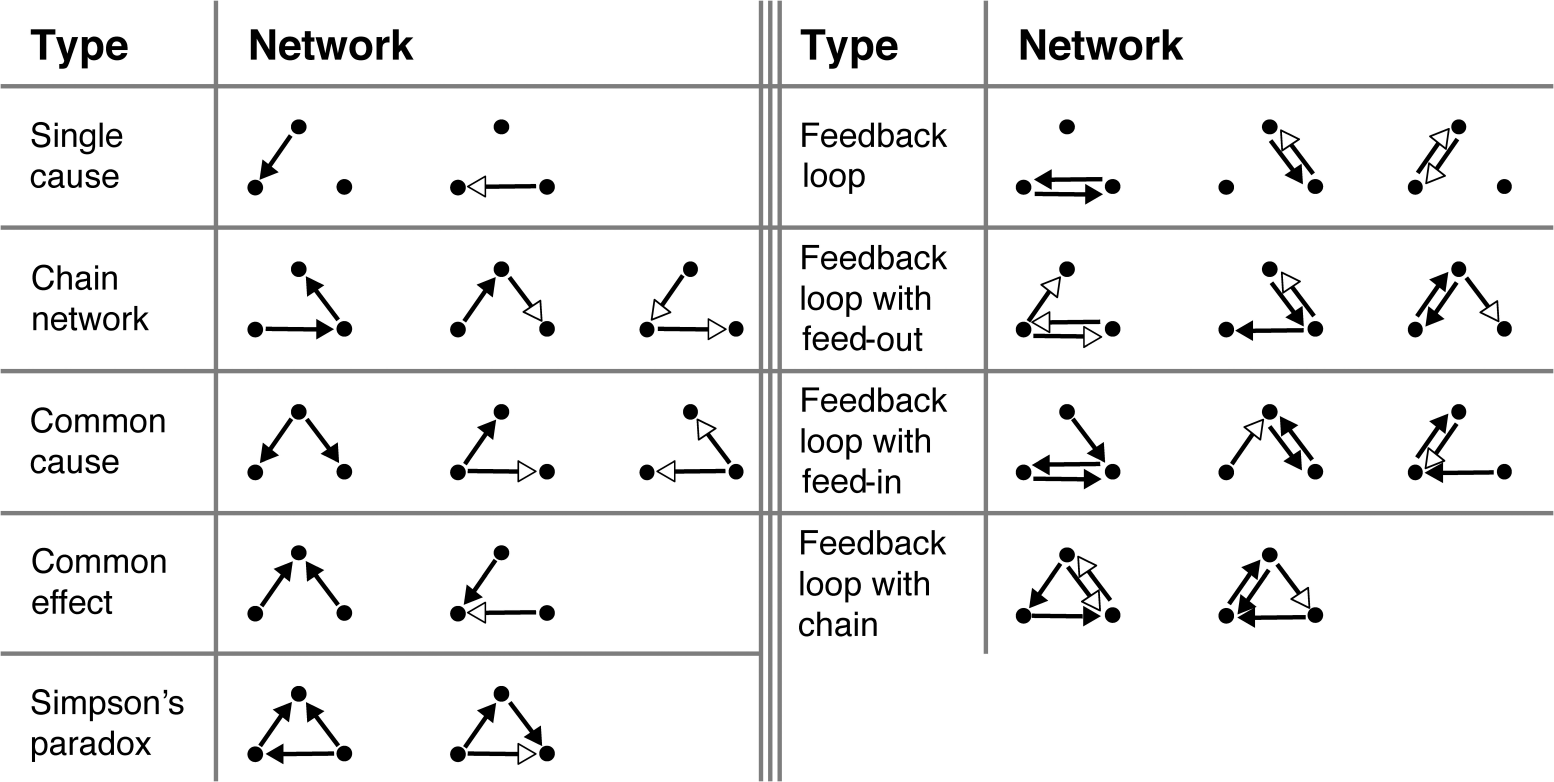
All 23 structures participants were tasked with learning. Black arrowheads signify “regular” connections (θ = 1), white arrowheads signify “inverse” connections (θ = −1).

The 23 causal graphs shown in Figure 5 were selected for testing on the basis of a number of criteria. They were roughly balanced in the number of positive and negative links and the number of links between each of the variables. More qualitatively, we tried to select networks that would be interesting a priori. This includes many of the classic causal graphs, such as chain networks, common causes, and common effects, but also less-studied graphs, such as those with feedback loops. The experiment always began with two practice trials that were excluded from all analyses. These were always the two *Single cause* networks (Figure 5, top left). This was followed by 23 test trials, one for each of the networks in Figure 5 presented in random order. The OU parameters used during training and the test were ω = 0.1 and σ = 5. The true θs were either 1 (for regular connections), 0 (no connection), or −1 (for inverse connections).

#### Procedure

To familiarize them with the interface, participants were required to first watch four videos of an agent interacting with example causal networks. These videos informed participants of the underlying causal structure and demonstrated an agent interacting with the system. To minimize biasing participants toward any particular intervention strategy, the videos displayed a variety of basic movements, including wobbling the intervened on variable, holding a variable at a constant level, and holding a variable at a limit value (e.g., 100) by moving its slider to one end of the scale. The four example causal networks included (1) no causal connections, (2) a single regular (θ = 1) connection, (3) a single inverse (θ = −1) connection, and (4) two connections forming a causal chain in which one link was regular and one was inverse. To ensure that they understood the task, participants were required to pass a five question comprehension check before starting. If a participant responded incorrectly to any of the five questions they were permitted to retake the quiz until they responded correctly to all five questions. This was designed to ensure that they learned: the duration of each trial, the difference between a regular and inverted connection, that there can be more than one connection per network, and that they must provide a response for all six possible connections.

**Figure 6:**
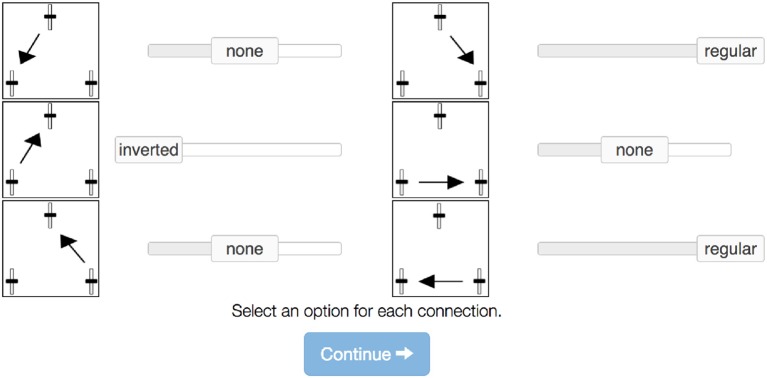
Judgment options for participants. Participants were presented with a ternary choice between “inverted,” “none,” and “regular”.

In the main task that followed, participants completed 25 trials lasting 45 s each. The first two of these involved a single regular and single inverse connection that, unknown to participants, we considered practice trials to familiarize them with the interface and excluded from all analyses. A trial was initiated by pressing the “Start” button, whereupon the sliders started moving with values updating every 100 ms. Perceptually, they would appear to “jitter” according to the noise associated with the underlying OU network plus move systematically according to the unknown causal relationships. At any time, participants were free to intervene on any variable by clicking, holding, or dragging the requisite slider. While it was pressed down, the position of the mouse determined the value of the variable. Once it was released the variable would continue from that point according to the OU network. Participants were free to make (and revise) their judgments at any point after initiating a trial but were required to enter a judgement for all six causal relations by the end of the trial (see Figure 6). No feedback was provided at any point. After completing the 25 trials, participants completed a brief post-test questionnaire reporting their age, gender, engagement and subjective difficulty as well as any comments.

### Results

Participants were substantially above chance (0.33) in correctly classifying causal links into one of the three response categories (*M* = 0.82, *SD* = 0.22), *t*_(29)_ = 17.48, *p* < 0.001. They were slightly more successful in identifying regular causal links (*M* = 0.92, *SD* = 0.12) than inverse causal links (*M* = 0.90, *SD* = 0.13), *t*_(29)_ = 2.12, *p* = 0.04. Participants also correctly classified a higher proportion of causal relationships as the trials progressed, as demonstrated by a simple linear regression of accuracy on trial number, *t*_(21)_ = 2.91, *p* = 0.008, although this relationship was modest with participants being 0.25% more likely to correctly identify a link for each new trial.

In identifying overall causal networks (correctly identifying all six of the possible directional causal relationships), participants were also well above chance (3^−6^ = 0.0014), (*M* = 0.44, *SD* = 0.22), *t*_(29)_ = 10.81, p < 0.001. The probability of selecting the correct network was 0.79, 0.60, 25, and 0.07 for networks with 1, 2, 3, and 4 causal links, respectively. Accuracy varied sharply with the complexity of model as shown by a repeated measures ANOVA, *F*_(3, 84)_ = 74.0, p < 0.001. Note that participants' responses did not reflect a preference toward simpler models, as they marked slightly over half of the possible connections (*M* = 0.52, *SD* = 0.13), which was greater than the true proportion of connections in the test networks (0.39), *t*_(29)_ = 5.62, p < 0.001. See the Supplementary Material for results for all tested networks.

#### Errors

While participants were generally well above chance in identifying causal relationships, there was some systematicity to their errors. In particular, these errors closely followed the qualitative predictions of Fernbach and Sloman (2009) local computations (LC) model. The first qualitative prediction is an over-abundance of causal links. Eighty-two percent (*SD* = 0.17) of the errors that participants made involved adding causal links that didn't exist, significantly greater than chance^4^ (0.59); *t*_(29)_ = 7.33, *p* < 0.001. The second qualitative prediction of the LC model as defined in this paper is an inability to distinguish between direct and indirect causes (e.g., in the network *X* → *Y* → *Z*, incorrectly also judging *X* → *Z*). While in general participants correctly classified 82% of the causal links, they were far more likely to erroneously add a direct link between two variables when in fact the relationship between those variables was mediated by a third variable, with below chance (0.33) accuracy on those potential links (*M* = 0.16, *SD* = 0.21);*t*_(29)_ = −4.48, *p* < 0.001.

**Figure 7:**
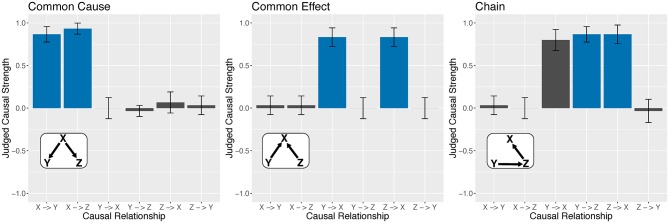
Participant judgments of causal relationships for three tested networks. Bar colors correspond to the true causal structure, namely, blue for regular connections and gray for no connection. Bar heights represent mean θ reported by participants (regular = 1 and none = 0). Because these networks included only regular causal relationships, no instances of inverse relationships are shown. Error bars Denote 95% confidence intervals.

Figure 7 shows participant judgments for three classic causal structures in causal cognition: common cause, common effect, and chain networks. It shows that participants were quite good at detecting any causal relationship in a network that existed between two variables. In the figure, these results correspond to the blue bars, which indicate that they correctly classified a regular connection as regular (as mentioned, participants were also good as classifying inverse connections as inverse). Figure 7 also shows that participants were often good at classifying absent connections as absent (the gray bars) with one important exception: in the chain network *Y* → *Z* → *X* the relationship between *Y* and *X* was judged to be nearly as causal as *Y* → *Z* and *Z* → *X*. That is, they failed to appreciate that the (apparent) relationship between *Y* and *X* was in fact mediated by *Z*. These patterns held for the other instances of the common cause, common effect, and chain networks defined in Figure 7. Moreover, we found that, for any of the more complex networks in Figure 7, participants had a strong tendency to infer a direct causal relationship between two variables whenever those variables were in fact mediated by the third variable. Figure S1 presents how causal links were classified for all 23 networks.

#### Interventions

To achieve this level of performance, participants made heavy use of interventions. We define a single intervention as beginning when a participant clicked on a variable's slider and ending when the mouse was released. The average number of interventions made on a single trial was 4.94 (*SD* = 2.46). However, because a few participants made a large number of interventions on most trials, this distribution was modestly skewed with a median of 4 and mode of 3. One participant made no interventions at all.

Interventions lasted an average of 3.46 s (*SD* = 3.00) and had a range (the maximum value of the variable during the intervention subtracted from its minimum value) of 138.3 (*SD* = 58.89). This latter measure was strongly bimodal with modes around 100 and 200, indicating that interventions typically consisted of participants dragging a variable from about 0 to one end of the scale (−100 or 100) or then in addition dragging it to the opposite end of the scale. Apart from these large swings, participants typically held the variable steady at a constant value during an intervention. This conclusion is supported by the fact that, within an intervention, the percentage of 100 ms time windows in which the variable had the same value as during the previous window was 71.2%. Four participants had some tendency to “wiggle” the variable through a small range during an intervention but they were the exception.

The interventions were spread about evenly over the three variables. Indeed, all three network variables were manipulated at least once on more than 99% of the trials. Interventions varied modestly as a function of whether the manipulated variable was a cause of other variables in the network. When it was, the intervention was both shorter (3.21 s) and had a narrower range (132.9) than when it wasn't (3.99 s and 149.5), *t*_(28)_ = 3.19 and *t*_(28)_ = 6.39, respectively, both *p*s < 0.005^5^. Apparently, it was easier for participants to identify causes, which involves observing a state change in other network variables, than non-causes, which involves the absence of such changes. Interventions on causes did not vary substantially, in length of time or range of values, as a function of whether they had one or two effects. Interventions also did not vary as a function of whether or not the variable was affected by other variables in the network. In summary, participants recognized that interventions help causal learning, that manipulating all variables is necessary to identify the correct causal structure, and that large interventions are more useful than small ones.

#### Results Summary

Participants exhibited considerable ability to intervene effectively and learn causal structure in our task. Despite these abilities, they also made systematic errors consistent with the predictions of the LC model. It is not clear whether the data considered as a whole is more consistent with normativity or a more locally focused model. Indeed, it is not even clear that participants are using the OU functional form to infer connections, rather than a more general model, such as one that assumes linearity. For a more granular analysis of people's causal structure learning, we now turn to a number of theoretical accounts of how people learn causal structure.

## Modeling

In this task we compare a total of nine models corresponding to different accounts of how people learn causal structure. These accounts can be roughly categorized as modeling people as normative, local, linear, or random in their causal learning behavior. We compare the ability of these models' to predict participants' causal structure judgments.

### OU Models

#### Normative Model

Normative inference for the current task requires that a learner maintain a distributional belief over all possible causal structures and update it according to the data they experience. Equation (6) above defines normative inference in this task. There has been much work suggesting that adults and children are capable learners of causal structures and act roughly in accordance with the normative model, at least in sufficiently simple scenarios (Gopnik et al., 2004; Griffiths and Tenenbaum, 2009). We ask whether these conclusions generalize to the sort of causal systems under investigation here.

Recall that Equation (6) assumes that learners have priors over ω, σ, and the θs. We assume for simplicity that learners acquire a rough approximation of the true values of these parameters [i.e., ω = 0.1, σ = 5, and θ ∈ (−1, 0, 1)] while watching the four instructional videos, but assume some spread to accommodate uncertainty. The distributions we assumed over parameters were thus^6^

$$\theta \sim \Gamma \left(\text{shape}=5\times \theta_{true},\text{ rate}=5\right)$$

$$\omega \sim \Gamma \left(\text{shape}=100\times \omega_{true},\text{ rate}=100\right)$$

$$\sigma \sim \Gamma \left(\text{shape}=100\times \sigma_{true},\text{ rate}=100\right)$$

Note that θ values are defined by the graph. For regular connections, θ is distributed as above. For inverse connections, the sampled values are negated. For non-connections θ is 0.

#### Local Computations Model

We compare the normative model to a “local computations” (LC) model that has been advocated as a general-purpose account of causal learning behavior (Fernbach and Sloman, 2009; Bramley et al., 2017a). Applied to an OU network, the LC model entails deciding, for each potential causal relationship considered in isolation, whether the observed values of those two variables implies a regular, inverted, or zero causal relation. It thus involves applying Equation (5) above to each potential causal relationship. The LC model assumes the same priors over ω, σ, and the θs as the normative model.

A key distinction between the normative and LC models of course is their ability to detect whether a relationship between two variables is mediated by a third. For example, in the network *X* → *Y* → *Z*, *X* and *Z* have many of the hallmarks of a direct causal relationship: They are correlated, changes in *X* precede changes in *Z*, and intervening on *X* later affects *Z* (but not vice versa). Whereas, the normative model would take into account the mediated relationship between *X* and *Z* (by noting the absence of an *X*/*Z* correlation when controlling for *Y*), LC, which evaluates individual causal links without consideration of the entire graph, would not recognize the mediating role of *Y* and so infer *X* → *Z* in addition to *X* → *Y* and *Y* → *Z*. Of course, we have already seen partial evidence that participants may be poor at detecting mediated relationships (Figure 7). Modeling will reveal whether the LC model is a good account of all the data, or if it only accounts for participants' errors.

### Alternative Models

We compare the two OU-based models to alternatives that assume linear relationships between cause and effect. In particular, we compare two approaches to modeling timeseries information from the literature: time-lagged correlation and Granger causality. Each of these approaches is applied to three candidate representations for learning causal structure between continuous variables, as introduced by Soo and Rottman (2018); *state representations, difference scores*, and *trinarized difference scores*.

In these linear models, the value of variable *i* at time *t* is modeled as

(7) $$\begin{array}{l} P\left(v_{i}^{t}|v^{t-1},\sigma ,\beta \right)=\underset{j}{\sum }[\beta_{ji}\cdot v_{j}^{t-1}]+N\left(0,\sigma \right) \end{array}$$

where *j* denotes all causes of variable *i* (including *i* itself) and β_*ji*_ denotes the partial slope coefficient or strength of that cause on the effect. Analogously to our treatment of θ values in the OU models, for the linear models we assume some uncertainty about the strength parameter *p*(β) but that these differ in sign for regular and inverse connections, and also model people as having uncertainty over standard deviation *p*(σ). The marginal likelihood of *v*_*i*_ for a graph thus involves computing, for each timepoint, the likelihood of that variable's value given the β predictors defined by the graph and the value(s) of its cause(s), and marginalizing over *p*(β) and *p*(σ). We treat interventions in the same manner as the OU models. As before, we compute the total likelihood as the product of the marginal likelihoods of all variables at all timepoints under each graph, assume an initially uniform prior over graphs and compute the resulting posterior. The unnormalized posterior probability of a causal graph given all values of all variables at all timepoints is thus

(8) $$\begin{array}{r} P\left(g_{k}|v;\iota \right)\propto \underset{t}{\prod }\underset{i}{\prod }\int_{\beta }\int_{\sigma }P\left(v_{i}^{t}|v^{t-1},\sigma ,\beta ;\iota_{i}\right)P\left(\beta |g_{k}\right)\\ P\left(g_{k}\right)P\left(\sigma \right)\text{ d}\sigma \text{d}\beta \end{array}$$

This general procedure can be applied to each of the linear models by modifying the state representation *v* or prior over β. For the three candidate representations introduced by Soo and Rottman (2018): State representations involves inference over the actual variable values; difference scores involves inference over variable values after computing *v*^*t*^ − *v*^*t* − 1^; trinarized difference scores involves inference over difference scores that have been converted to −1 when negative and 1 when positive.

The difference between time-lagged correlation and Granger causality is just whether β_*ii*_ is included as a predictor, that is, whether $v_{i}^{t}$ is influenced by $v_{i}^{t-1}$ as well as its causes. Granger causality includes this term while Time-lagged correlation does not.

Unlike the OU models, there is no natural ground truth parametrization for the linear models on which to center reasonable distributional parameter beliefs. Thus, we must find another way to choose reasonable settings for *p*(β) and *p*(σ). We chose the mean of our distributions by fitting the ${\hat{\beta }}_{ii}$, ${\hat{\beta }}_{ji}$, and $\hat{\sigma }$ values that maximized the posterior probability of the true causal graphs across all subject data (including β_*ii*_ for the Granger models). We then made analogous assumptions about the spread around these means as we did for θ and σ in the OU models—namely,

(9) $$\begin{array}{r} \beta \sim \Gamma \left(\text{shape}=5\times \hat{\beta },\text{ rate}=5\right)\\ \sigma \sim \Gamma \left(\text{shape}=100\times \hat{\sigma },\text{ rate}=100\right). \end{array}$$

β values are treated the same as in the OU models. Regular connections are distributed as above, inverse connections are negated.

### Comparing the Models

We compare participants' structure judgments to the predictions of these models across all the test trials in our experiment. In total, we consider nine models. These are eight described above: (1) *normative*, (2) *local computations (LC)*, and three variants of both (3–5) *Granger causality* and (6–8) *Time lagged correlation* varying whether they were based directly on states, difference scores, or trinarized difference scores. Finally, we compare these against (9) a *Baseline* model that assumes each judgment is a random selection from the space of possible graphs. We marginalized over θ, ω, σ by drawing 1,000 samples from their respective distributions and averaging the likelihood within each causal model. To account for decision noise in selecting causal graphs from their posterior distributions, for each model apart from the baseline we fit (by maximum likelihood using R's optim function) a single softmax parameter τ that maximized the posterior probability of participant selections.

#### Results and Discussion

Table 1 details the results of our comparison. For each inference model we report the overall proportion of the true connections identified across all trials assuming the most probable graph is selected at the end of each trial (Accuracy column), the proportion of participant's edge judgments that correspond with the most probable graph under the model (Judge column), the Bayesian Information Criterion of all participant's judgments according to that model (BIC column); and the number of participants best fit by each model^7^.

**Table 1:** Summary of model accuracy and performance.

	**Model**	**State representation**	**Accuracy**	**Judge**	**BIC**	**Px**
1	OU local computations		0.89	0.82	6,163	21
2	OU normative		1.00	0.82	6,475	4
3	Granger causality	States	0.91	0.78	7,079	1
4		Difference scores	0.82	0.69	8,415	1
5		Trinarized diff scores	0.49	0.42	9,859	0
6	Time-lagged correlation	States	0.89	0.74	7,901	1
7		Difference scores	0.82	0.69	8,407	0
8		Trinarized diff scores	0.63	0.50	9,793	0
9	Baseline		0.17	0.17	9,888	2

*Accuracy, proportion of links drawn that match ground truth; Judge, proportion of links drawn that match participant judgments; BIC, Bayesian Information Criterion; Px, number of participants best fit by that model*.

Unsurprisingly, the normative model was the most successful at recovering the underlying structure, but many other models were also successful. The only models that struggled were those that used trinarized difference scores as their representation, showing that the magnitude of changes in the variables is important to capturing the structure of the data.

Next, we compared the maximum *a posteriori* estimates of causal structure of the models to participant judgments. In this coarse measure, the OU models were roughly equal to each other in matching participant judgments, and were also similar to some of the linear models.

The results of the more sensitive posterior probability analysis were clearer in distinguishing between models. Over all participants, the LC model had the highest log-likelihood. On a per participant basis, of the 30 participants 21 were best fit by the LC model, with the normative model being the best account of four participants. The remaining five participants were split among the linear models or were at baseline.

## General Discussion

In this paper, we introduced a generative model of causal influence relating continuous variables over time. We showed how such systems can exhibit emergent behaviors, such as excitatory or inhibitory feedback and oscillations, depending on specific settings of relative causal strengths between variables. When learning from this rich data, people were best described as considering individual pairs of variables, rather than updating their beliefs over entire structures. This finding accords with an intuitive description of how people handle continuous information flowing in real time: they focus their attention on smaller, more manageable problems rather than attempting to tackle the full torrent of information.

### Local Inference

A key result in our task was that most participants evaluated pairwise relationships between variables rather than updating their beliefs over all possible causal structures. This conclusion was drawn from the superior fit of the locally focused LC model, and corroborated by qualitative results, such as the finding that participants often inferred direct causal relationships between variables that were in fact only indirectly related (through a third mediating variable). These results are consistent with previous findings suggesting that, rather than representing a full hypothesis space, people tend to consider a single hypothesis to which they make small alterations (Quine, 1960; Fernbach and Sloman, 2009; Bramley et al., 2017a). Here we show that this principle of causal learning extends to much richer scenarios. Indeed, it may be the case that real time continuous information places stronger demands on attention and memory than the original settings that provided evidence for the LC model. If this were true, it would be especially reasonable to use the resource-efficient local strategy in these more demanding environments.

A potential alternative conceptualization of the LC model is that it instantiates the idea that distal causes are still considered as causal. For example, most people would not find it inappropriate to say that the reintroduction of wolves to Yellowstone National Park caused changes to the ecosystem, even if many of these changes came indirectly through other variables, such as changes in the movement of elk (Fortin et al., 2005). While this is a reasonable conceptualization, we believe that it is not as good an account of our data as the LC model. For one, we explicitly provided participants with an example in the instructions that showed the movement of a chain network without the additional indirect connection. This should have reduced the possibility that participants were unclear about whether they should consider distal causes as causal. This accords with findings in the literature that people exhibit locality despite feedback, incentives, and explicit instruction with examples that encourage people to not draw the additional causal link (Fernbach and Sloman, 2009; Bramley et al., 2015, 2017a). More fundamentally, this “distal” account makes assumptions about how people are approaching the task that we consider unlikely. It models them as doing full normative inference, and then having a response bias to draw indirect connections. Figure S1 shows that indirect connections were less likely to be responded to as causal than the direct connections, which would imply a response bias where participants have the full causal model but would only on occasion draw the additional indirect connection. The LC model, in contrast, naturally considers indirect connections as less causal due to the underlying dynamics of OU networks. While indirect causal relationships do have many hallmarks of direct causal relationships (correlation, temporal asymmetry, asymmetric results of interventions), they are not identical. In *X* → *Y* → *Z*, changes to *Z* in response to *X* are more temporally removed and noisier than would be predicted if there were a direct *X* → *Z* connection, and therefore the LC model assigns a lower (but still reliably non-zero) probability to these potential connections. Because the LC model accounts for the patterns of errors as naturally arising from the interaction of system dynamics and cognitive limitations, rather than as a response bias over normative inference, we consider it a better account of the behavior of participants in our task.

### Interventions

One contribution of the OU network framework is the introduction of a qualitatively different type of intervention. In a typical study of causal cognition learners are able to, on a particular trial, turn a variable on or off and observe the values of other variables. In contrast, interventions in our task are extended through time and can encompass a wide range of variable values. Participants generally recognized that the most informative actions involved large swings in variable values and systematic manipulation of each variable in the system^8^.

Nevertheless, note that while their interventions were informative they were less than optimal. In fact, the most efficient interventions in this task involve rapid swings between the ends of the variable's range. But whereas participants used the full range, they tended to hold a variable at one value for longer than necessary. Doing so yields useful but somewhat redundant information. Of course, perhaps this strategy reflected participants' need for redundant information imposed by cognitive processing limits. It may also reflect their inability or unwillingness to engage in the rapid motor movements required by the optimal strategy.

Although participants could intervene on any variable at any time to set it to any value, they were constrained to manipulating one variable at a time. Future studies could expand the action space by, for example, allowing participants to “freeze” one variable at a value while manipulating others. Of course, an ability to “control for” one variable while investigating the relationship between two others might help learns identify mediating relationships. For example, freezing *Y* and then manipulating *X* in *X* → *Y* → *Z* would result in to no change in *Z*, perhaps reducing the chance that the learner would conclude *X* → *Z*. This approach could be considered an application of learning strategies from the CPS literature to environments without sharp distinctions between input and output nodes (Kuhn and Brannock, 1977; Schoppek and Fischer, 2017), with the additional information generated by the “Do()” operator's graph surgery.

### Future Directions

The proposed OU network framework can be extended across a variety of dimensions in future research. For example, in this paper's instantiation of OU networks, a cause impacts an effect on the next timepoint. The impact of a cause on effect could be distributed over multiple timepoints, or at some stochastically selected timepoint. Such studies could contribute to debates about the influence of time on causal learning, such as that judgments of causality are strengthened by temporal contiguity (Shanks et al., 1989) or the reliability of delays (Buehner and May, 2003; Bramley et al., 2018). Varying the gap between timepoints (in this task *t* to *t* + 1 was 100ms) may result in different approaches by participants. Use of continuous variables naturally allows consideration of a greater number functional forms relating causes and effects (Griffiths and Tenenbaum, 2009). Latent causes can be introduced to model implicit inference of mechanisms relating cause and effect. Complex, non-linear data can be generated to study people's learning from time series data (Soo and Rottman, 2018; Caddick and Rottman, 2019). The outcomes of experiments using these richer causal systems will help to evaluate the generalizability of models of causal cognition that have heretofore been tested mostly on Bayes nets applied to discrete events.

The formalism developed in this paper also has potential application to the domain of control. Many aspects of everyday life, as well as interesting domains in AI and machine learning, can be can be classed as control problems in which there is initial or ongoing uncertainty about the structure of the control domain. As discussed in the introduction, there is an extensive literature known as Complex Problem Solving that has participants manipulate environments that are reactive to their decisions to maximize gain (for review, see Osman, 2010). One limitation of extant work is that they do not include learning models that can help distinguish between learning and control performance. In parallel, much recent attention in machine learning has been given to demonstrations of successful control in small worlds, such as atari and board games. However, generalization to new goals or related environments continues to be poor (Lake et al., 2017). In recent work, we propose OU networks as a systematic class of control environments. This approach allows research into human control to ask new questions, such as what structures are inherently easy or hard to identify or control and under what circumstances does successful control depend on an accurate model of a system's structure (Davis et al., 2018).

### Functional Form

Given people's well-known bias toward assuming linear functional forms (Brehmer, 1974; Byun, 1996; DeLosh et al., 1997; Kalish et al., 2004, 2007; Kwantes and Neal, 2006), it may be a surprising result that the alternative models assuming linearity did not match people's judgments as well as those using the Ornstein–Uhlenbeck functional form. This result has a number of possible explanations. For one, as discussed before, Ornstein–Uhlenbeck processes appear to be relatively common across a range of domains, and people may have a developed representation of the functional form that they brought to the task. It is also possible that participants do not have a direct representation of Ornstein–Uhlenbeck processes, but were able to recognize higher-order movement statistics that are not present in linear models (e.g., OU processes, unlike linear relationships, exhibit acceleration toward their attractor basin). For example, people may have applied a general function approximator, such as a Gaussian Process to the relationship between cause and effect and abstracted a function closer to OU processes than linearity. Future work could explore settings where learning the functional form between cause and effect is not possible (such as one-shot learning) or settings where the impact a cause has on its effect is linear.

### Limitations

There are a number of limitations to the current project that could be addressed with further experiments. For one, while we did account for uncertainty over parameters of our models, we did not account for other sources of noise, such as the likelihood that people cannot attend to all three variables simultaneously^9^. This issue will likely compound as more variables are added. Additionally, the presented analyses in this paper discuss but do not model intervention decision-making, a critical component of the active learning of causal structure. Future analyses would naturally involve, as a benchmark to compare against humans, models for selecting actions that maximize expected information gain. This information maximizing strategy could be compared to other strategies from the Complex Problem Solving literature that involve changing a single variable at a time (Kuhn and Brannock, 1977; Schoppek and Fischer, 2017).

### Conclusions

We have no doubt that the canonical causal relationships between discrete events (e.g., take a pill → headache relieved) that have been the main focus of causal cognition often serve as highly useful and approximately correct parts of human's semantic representation of the world. But sometimes details matter. Causal influences emerge over time, may reflect functional relationships that are as complex as the underlying mechanisms that produce them, and afford interventions that vary in their duration and intensity. Complex patterns of feedback may be the rule rather than the exception (Cartwright, 2004; Strevens, 2013; Sloman and Lagnado, 2015). Apprehending these properties may even be a precondition to forming the (highly summarized and approximate) causal relations between discrete events that are so simple to represent and easy to communicate.

We instantiated a learning task in which people were confronted with some of these challenges, including continuously-observed continuous variables, feedback cycles, and the ability to carry out extended interventions. We found that they exhibited considerable success identifying the correct causal structure but also committed systematic errors, errors consistent with a model that describes people as narrowly investigating individual causal relationships rather than updating their beliefs wholesale. We hope that the formalism presented in this paper will be help spur greater study of the mechanisms for learning and action in this important class of problems.

## Data Availability Statement

Raw data is available at the public website: https://zach-davis.github.io/publication/cvct/.

## Ethics Statement

The studies involving human participants were reviewed and approved by New York Institutional Review Board. The patients/participants provided their written informed consent to participate in this study.

## Author Contributions

All authors listed have made a substantial, direct and intellectual contribution to the work, and approved it for publication.

### Conflict of Interest

The authors declare that the research was conducted in the absence of any commercial or financial relationships that could be construed as a potential conflict of interest.

## Footnotes

^1^Throughout this work we use subscripts to denote variables and superscripts to denote time. Note that whereas $v_{i}^{t}$ is the value of *i* at time *t*, *v*_*i*_ is the value of *i* at all timesteps, *v*^*t*^ is the value of all variables at time *t*, and *v* is the value of all variables at all times.

^2^Although the OU formalism allows it, throughout this work we ignore the possibility of self-cycles, that is, instances in which variables is a cause of itself. That is, we assume, Θ_*ii*_ = 0.

^3^See https://zach-davis.github.io/publication/cvct/ for a demo.

^4^For the structures used in this experiment, a hypothetical participant who responded “inverse,” “none,” and “positive” with equal probability would erroneously add a causal link 59% of the time.

^5^There were 28° of freedom for these analyses, rather than 29, because one of the 30 participants did not intervene.

^6^The 5 and 95% quantiles associated with these distributions are 0.39 and 1.83 for θ, 0.054 and 0.157 for ω, and 4.64 and 5.37 for σ. These variances were chosen to accommodate a moderate amount of uncertainty in beliefs about each of the higher-level parameters, while still being consistent with the qualitative behavior of the system under the true parameters.

^7^A *post-hoc* power test was computed for the null hypothesis that the number of participants best fit by each of the nine models would be equally distributed. For a chi-squared test with 30 participants, 8° of freedom, and the observed effect size of 1.91, the probability of observing an α < 0.05 is 0.999.

^8^The observed systematic strategy of manipulating a single variable, holding it at a value, and observing the downstream effects closely corresponds to successful learning strategies from the CPS literature, such as VOTAT and PULSE.

^9^Although Vul et al.'s (2009) finding that people optimally allocate attention to particles moving according to an OU process may ameliorate the latter concern.
