# Supplementary material for: Causal Structure Learning in Continuous Systems
Source: Front Psychol. 2020 Feb 20;11:244. doi: 10.3389/fpsyg.2020.00244 (PMC7044349; doi:10.3389/fpsyg.2020.00244)
Supplement: Supplementary file 1 [file Image_1.pdf]

Supplementary Materials for "Causal Structure Learning in Continuous Systems"

Zachary J. Davis, Neil R. Bramley, Bob Rehder

February 13, 2020

Figure S1 presents results for all 23 tested networks. Note that although overall performance was good, participants consistently reported the presence of a direct causal relationship between variables whenever that relationship was in fact mediated by the third variable. For example, in the first instance of a Chain network in the figure ($Y \rightarrow Z \rightarrow X$), participants incorrectly judged that $Y \rightarrow X$ (i.e., ignored the mediator Z). In the first instance of a Feedback Loop W/Feedout ($Z \leftrightarrow Y \rightarrow X$), they judged that $Z \rightarrow X$ (ignoring mediator Y). In the first instance of a Feedback Loop W/Feedin ($X \rightarrow Z \leftrightarrow Y$), they judged that $X \rightarrow Y$ (ignoring mediator Z). In the first instance of a Feedback Loop W/Chain ($Y \rightarrow Z \leftrightarrow X \rightarrow Y$), they judged that $Y \rightarrow X$ (ignoring mediator Z) and $Z \rightarrow Y$ (ignoring mediator X).

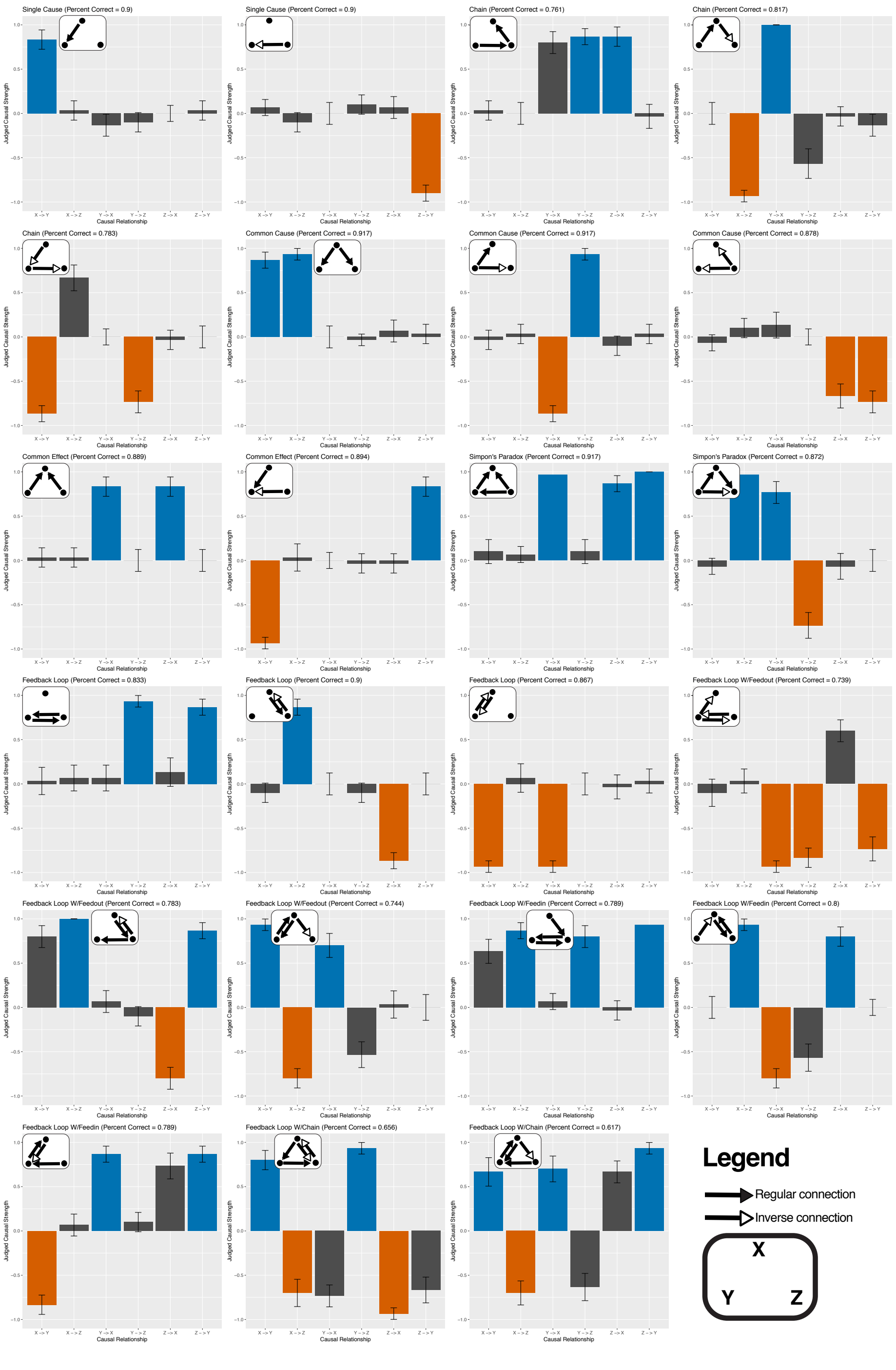

Figure S1: Participant judgments of causal relationships for all 23 tested networks. Blue, gray, and red bars correspond to regular, absent, and inverse connections, respectively. Bars represent the mean θ reported by participants, where regular $= 1$, none $= 0$, and inverse $= -1$. Error bars denote 95% confidence intervals.
